# Supplementary material for: Genome-Wide Identification of the Paulownia fortunei Aux/IAA Gene Family and Its Response to Witches’ Broom Caused by Phytoplasma
Source: Int J Mol Sci. 2024 Feb 13;25(4):2260. doi: 10.3390/ijms25042260 (PMC10889751; doi:10.3390/ijms25042260)
Supplement: Supplementary file 1 [file ijms-25-02260-s001.zip › Supplementary figures.pdf]

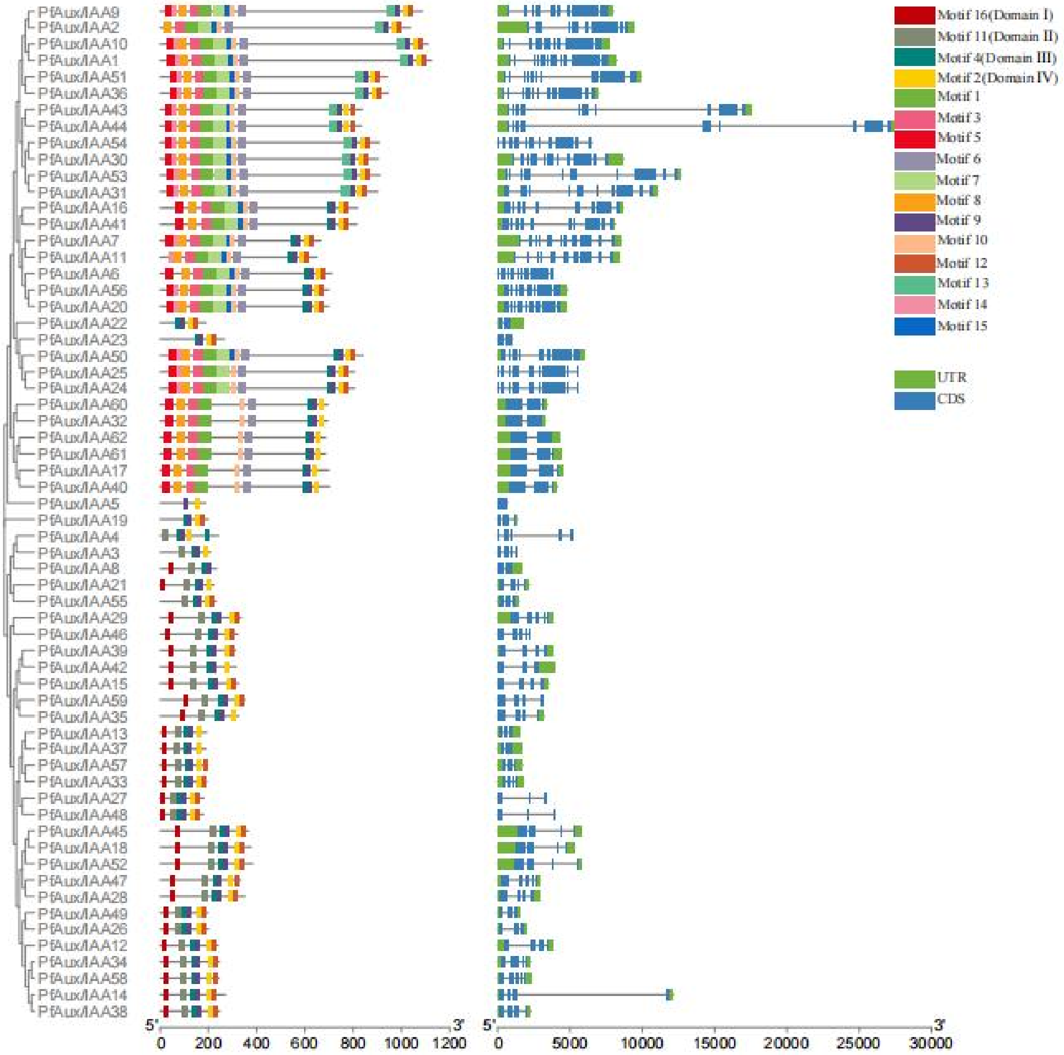

Figure S1. Conserved motifs and gene structure of *Aux/IAA* gene family in *P. fortunei*.

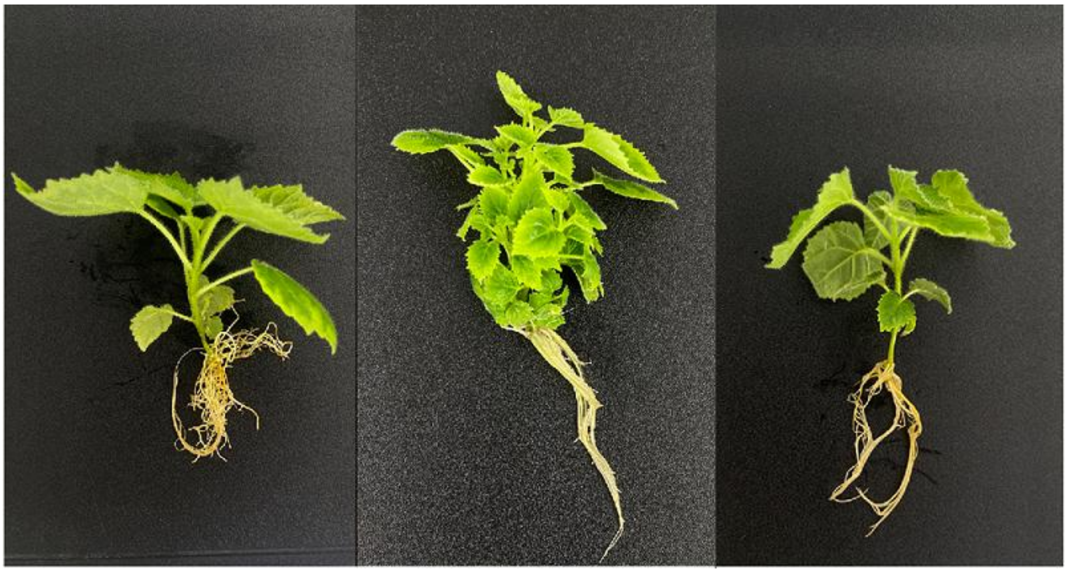

Figure S2. The phenotype changes of PaWB-diseased seedlings after MMS (20 mg·L^{-1}) treatment, from left to right, it is PF, PFI, PFI+MMS.

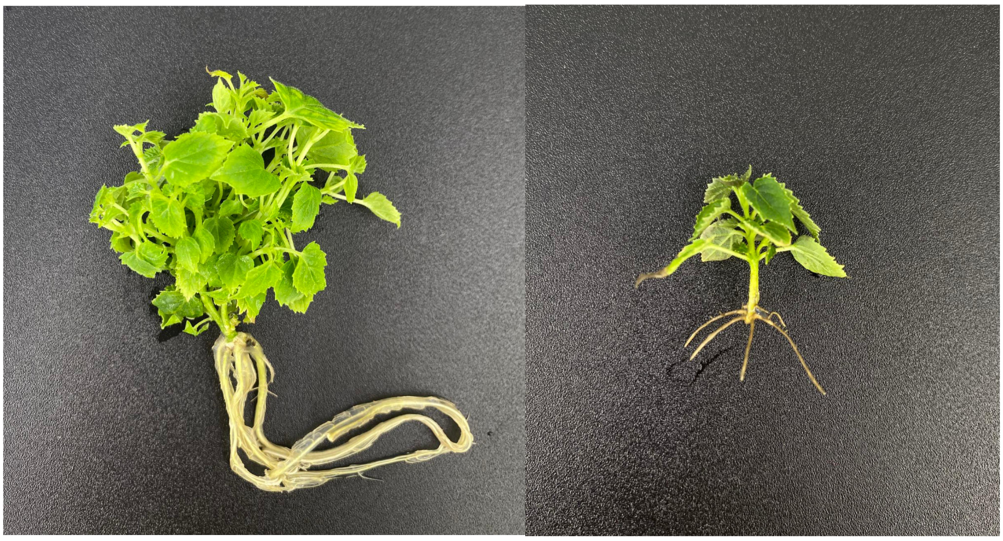

Figure S3. The phenotype changes of PaWB-diseased seedlings after SA (13.8 mg·L^{-1}) treatment, from left to right, it is PFI, PFI+SA.
