# Supplementary material for: Genome-Wide Identification of the Paulownia fortunei Aux/IAA Gene Family and Its Response to Witches’ Broom Caused by Phytoplasma
Source: Int J Mol Sci. 2024 Feb 13;25(4):2260. doi: 10.3390/ijms25042260 (PMC10889751; doi:10.3390/ijms25042260)
Supplement: Supplementary file 1 [file ijms-25-02260-s001.zip › Table S1.pdf]

Gene ID	Transcript ID	Protein ID	The number of amino acid	Molecular weight(KD)	pI	Instability index	Gravy	Subcellular localization
PfAux/IAA1	Pfo01g001280.1	KAI3443459.1	1124	124.086	6.18	62.87	-0.541	Nucleus
PfAux/IAA2	Pfo01g001920.1	KAI3443523.1	1037	115.752	6.15	65.93	-0.618	Nucleus
PfAux/IAA3	Pfo01g005530.1	KAI3443864.1	209	23.893	5.99	48.5	-0.665	Nucleus
PfAux/IAA4	Pfo01g005570.1	KAI3443868.1	242	27.177	8.49	38.4	-0.412	Nucleus
PfAux/IAA5	Pfo01g008610.1	KAI3444160.1	188	21.066	7.83	60.4	-0.481	Nucleus
PfAux/IAA6	Pfo02g000540.1	KAI3444396.1	712	79.862	7.27	52.65	-0.494	Nucleus
PfAux/IAA7	Pfo02g012360.1	KAI3445548.1	666	74.588	5.83	63.84	-0.529	Nucleus
PfAux/IAA8	Pfo03g005620.1	KAI3446896.1	234	26.636	7.97	45.04	-0.254	Nucleus
PfAux/IAA9	Pfo03g009180.1	KAI3446896.1	1087	120.765	6.35	66.35	-0.589	Nucleus
PfAux/IAA10	Pfo03g009690.1	KAI3447292.1	1111	122.545	6.21	62	-0.537	Nucleus
PfAux/IAA11	Pfo04g005060.1	KAI3448378.1	650	73.101	6.17	63.77	-0.464	Nucleus
PfAux/IAA12	Pfo04g008340.1	KAI3448691.1	240	26.402	6.91	68.62	-0.584	Nucleus
PfAux/IAA13	Pfo05g004010.1	KAI3449712.1	193	21.611	5.96	61.66	-0.66	Chloroplast
PfAux/IAA14	Pfo05g004020.1	KAI3449713.1	270	30.552	5.38	64.96	-0.68	Chloroplast
PfAux/IAA15	Pfo05g005160.1	KAI3449826.1	325	34.502	9.11	56.07	-0.441	Chloroplast
PfAux/IAA16	Pfo05g012580.1	KAI3450531.1	818	89.979	6.11	57.39	-0.416	Nucleus
PfAux/IAA17	Pfo05g013000.1	KAI3450573.1	699	76.91	7.92	46.16	-0.336	Nucleus
PfAux/IAA18	Pfo06g009450.1	KAI3451857.1	378	4.067	6.24	46.64	-0.438	Nucleus
PfAux/IAA19	Pfo07g005230.1	KAI3453064.1	200	22.895	5.48	61.38	-0.392	Nucleus
PfAux/IAA20	Pfo07g013070.1	KAI3453842.1	669	77.921	6.24	56.91	-0.543	Nucleus
PfAux/IAA21	Pfo07g016780.1	KAI3454210.1	224	25.839	4.97	40.03	-0.792	Nucleus
PfAux/IAA22	Pfo08g009310.1	KAI3455144.1	190	21.256	5	49.96	-0.828	Nucleus
PfAux/IAA23	Pfo08g009340.1	-	267	29.684	5.43	54.66	-0.776	Nucleus
PfAux/IAA24	Pfo08g009330.1	-	807	90.351	6.87	64.03	-0.675	Nucleus
PfAux/IAA25	Pfo08g009320.1	KAI3455145.1	807	90.351	6.87	58.25	-0.675	Nucleus
PfAux/IAA26	Pfo08g011770.1	KAI3455385.1	201	22	5.23	55.28	-0.357	Nucleus
PfAux/IAA27	Pfo08g011780.1	KAI3455386.1	181	20.095	5.63	50.1	-0.468	Nucleus
PfAux/IAA28	Pfo08g012460.1	KAI3455454.1	352	38.026	7.59	73.1	-0.438	Nucleus
PfAux/IAA29	Pfo08g012860.1	KAI3455494.1	336	36.554	9.13	53.19	-0.636	Nucleus
PfAux/IAA30	Pfo09g002160.1	KAI3455967.1	904	99.507	6.23	64	-0.349	Nucleus
PfAux/IAA31	Pfo09g019300.1	KAI3457663.1	903	100.021	6.04	59.78	-0.432	Nucleus
PfAux/IAA32	Pfo10g012370.1	KAI3458975.1	695	76.706	6.07	40.75	-0.397	Nucleus
PfAux/IAA33	Pfo11g003030.1	KAI3459452.1	193	21.384	8.3	37.75	-0.617	Nucleus
PfAux/IAA34	Pfo11g003040.1	KAI3459453.1	246	27.094	7.56	66.63	-0.479	Nucleus
PfAux/IAA35	Pfo11g004110.1	KAI3459559.1	326	35.041	8.66	38.75	-0.209	Vacuole
PfAux/IAA36	Pfo11g012450.1	KAI3460380.1	945	104.596	5.27	55.7	-0.426	Nucleus
PfAux/IAA37	Pfo12g002100.1	KAI3460926.1	190	21.41	5.72	66.85	-0.702	Nucleus
PfAux/IAA38	Pfo12g002110.1	KAI3460927.1	247	27.492	6.35	63.56	-0.627	Nucleus
PfAux/IAA39	Pfo12g002900.1	KAI3460994.1	314	33.505	8.55	53.44	-0.399	Nucleus
PfAux/IAA40	Pfo12g007220.1	KAI3461425.1	702	77.463	7.03	46.5	-0.283	Nucleus
PfAux/IAA41	Pfo12g007490.1	KAI3461452.1	816	90.108	6.22	51.88	-0.438	Nucleus
PfAux/IAA42	Pfo12g011480.1	KAI3461833.1	316	33.859	6.5	50.46	-0.301	Chloroplast
PfAux/IAA43	Pfo13g003410.1	KAI3462267.1	838	93.289	5.85	57.09	-0.417	Nucleus
PfAux/IAA44	Pfo13g003420.1	KAI3462268.1	835	93.15	5.98	60.75	-0.491	Nucleus
PfAux/IAA45	Pfo13g007110.1	KAI3462627.1	365	39.097	8.31	69.48	-0.542	Nucleus
PfAux/IAA46	Pfo14g001490.1	KAI3463298.1	322	35.354	9.24	72.39	-0.673	Nucleus
PfAux/IAA47	Pfo14g001710.1	KAI3463320.1	334	36.2	7.51	48.28	-0.466	Nucleus
PfAux/IAA48	Pfo14g002100.1	KAI3463359.1	179	19.904	5.06	44.9	-0.554	Nucleus
PfAux/IAA49	Pfo14g002110.1	KAI3463360.1	198	21.951	6.4	72.32	-0.46	Chloroplast
PfAux/IAA50	Pfo14g003890.1	KAI3463537.1	842	93.877	5.91	57.51	-0.628	Nucleus
PfAux/IAA51	Pfo15g006350.1	KAI3464862.1	941	104.031	5.66	54.8	-0.385	Nucleus
PfAux/IAA52	Pfo15g009730.1	KAI3465192.1	384	41.695	8.65	70.1	-0.453	Nucleus
PfAux/IAA53	Pfo16g000590.1	KAI3465574.1	912	101.033	5.96	63.69	-0.426	Nucleus
PfAux/IAA54	Pfo16g013440.1	KAI3466849.1	908	100.397	6.48	69.73	-0.386	Nucleus
PfAux/IAA55	Pfo18g000450.1	KAI3468016.1	232	270.48	5.01	33.85	-0.698	Nucleus

PfAux/IAA56	Pfo18g006280.1	KAI3468589.1	699	78.172	6.16	59.5	-0.542	Nucleus
PfAux/IAA57	Pfo19g004880.1	KAI3469682.1	196	21.602	5.4	55.36	-0.599	Nucleus
PfAux/IAA58	Pfo19g004890.1	KAI3469683.1	245	26.929	6.22	72.08	-0.438	Chloroplast
PfAux/IAA59	Pfo19g006680.1	KAI3469859.1	352	37.426	8.79	29.81	-0.238	Vacuole
PfAux/IAA60	Pfo20g001420.1	KAI3470472.1	696	76.884	6.11	45.43	-0.354	Nucleus
PfAux/IAA61	Pfoxxg019210.1	-	686	75.156	7.59	46.34	-0.353	Nucleus
PfAux/IAA62	Pfoxxg026840.1	-	686	75.213	7.94	47.11	-0.373	Nucleus
