# Supplementary material for: Genome-Wide Identification of the Paulownia fortunei Aux/IAA Gene Family and Its Response to Witches’ Broom Caused by Phytoplasma
Source: Int J Mol Sci. 2024 Feb 13;25(4):2260. doi: 10.3390/ijms25042260 (PMC10889751; doi:10.3390/ijms25042260)
Supplement: Supplementary file 1 [file ijms-25-02260-s001.zip › Table S2.pdf]

Purpose	Primer	Sequence (5′→3′)
Full-Cloning	*PfAux/IAA45* -F	ATGTCGCCACCACAATTGGG
	PfAux/IAA45 -R	CTAGTTCCGGTTCTTAGATTTTTCCA
	PfARF13 -F	ATGAAATTCTCTGCTGGCTTAAATC
	PfARF13 -R	TCAGAAATCAAGAGTCCCAACAGA
qPCR	*PfActin* -F	AATGGAATCTGCTGGAAT
	PfActin -R	ACTGAGGACAATGTTACC
	PfAux/IAA13 -F	CGGATCCGGGATGTTTGTGA
	PfAux/IAA13 -R	TGAGTACACGCCTATGGTGC
	PfAux/IAA29 -F	TTCAACCAACCCTTGCGTTC
	PfAux/IAA29 -R	TTGTGGGTGGGCTGATTGAA
	PfAux/IAA33 -F	CCAAGGCACAGATAGTTGGGT
	PfAux/IAA33 -R	TGAGATAAGGGGCTCCATCCA
	PfAux/IAA37 -F	TAAGGCACAAGTGGTTGGCT
	PfAux/IAA37 -R	AGCTCCATCCATGCTCACTTT
	PfAux/IAA38 -F	GGTAAATGCGACGCACAAGG
	PfAux/IAA38 -R	TCCCCGACAAGCATCCAATC
	PfAux/IAA45 -F	TGGGTCGGGGTTGAAAGAAC
	PfAux/IAA45 -R	CCAGGAAGACCAAGCGTCAA
	PfAux/IAA58 -F	AGTTTGCCGGAATATGGGGG
	PfAux/IAA58 -R	ATGGGGTCACTTTCACCACC
Yeast Two-Hybrid assay	pGBKT7-PfAux/IAA45-F	TCAGAGGAGGACCTGCATATGATGTCGCCACCACAATTGGG
	pGBKT7-PfAux/IAA45-R	CCGCTGCAGGTCGACGGATCCCTAGTTCCGGTTCTTAGATTTTTCCA
	pGADT7-PfARF13-F	GCCATGGAGGCCAGTGAATTCATGAAATTCTCTGCTGGCTTAAATC
	pGADT7-PfARF13-R	CAGCTCGAGCTGATGGATCCTCAGAAATCAAGAGTCCCAACAGA
Bimolecular fluorescence complementation	ECN-PfARF13-F	AGTGGTCTCTGTCCAGTCCTATGAAATTCTCTGCTGGCTTAAATC
	ECN-PfARF13-R	GGTCTCAGCAGACCACAAGTGAAATCAAGAGTCCCAACAGA
	ENN-PfAux/IAA45-F	AGTGGTCTCTGTCCAGTCCTATGTCGCCACCACAATTGGG
	ENN-PfAux/IAA45-R	GGTCTCAGCAGACCACAAGTGTTCCGGTTCTTAGATTTTTCCA
